# Effect of knife castration on leukocyte cytokine expression and indicators of stress, pain, and inflammation in Korean cattle bull calves

**DOI:** 10.5713/ab.22.0368

**Published:** 2023-01-08

**Authors:** Seonpil Yoo, Seok-Hyun Beak, Hyeok Joong Kang, Da Jin Sol Jung, Dilla Mareistia Fassah, InHyuk Jeong, Seung Ju Park, Md Najmul Haque, Myunghoo Kim, Myunggi Baik

**Affiliations:** 1 Department of Agricultural Biotechnology and Research Institute of Agriculture and Life Sciences, College of Agriculture and Life Sciences, Seoul National University, Seoul 08826, Korea; 3 Department of Animal Science, College of Natural Resources and Life Sciences, Pusan National University, Miryang 50463, Korea; 4 Institutes of Green Bio Science Technology, Seoul National University, Pyeongchang 25354, Korea

**Keywords:** Castration Stress, Cytokine Gene Expression, Inflammation, Korean Cattle Calves

## Abstract

**Objective:**

This study investigated the effects of surgical castration on behavior, physiological and inflammatory indicators, and leukocyte cytokine mRNA levels in Korean cattle bull calves.

**Methods:**

Nineteen Korean cattle bull calves (average body weight, 254.5 kg; average age, 8.2 months) were divided into two treatment groups: control (n = 9) and castration (n = 10). Surgical castration was performed using Newberry knives and a Henderson castrating tool. Blood was obtained just before castration (0 h) and at 0.5 h, 6 h, 1 d, 3 d, 7 d, and 14 d after castration. Plasma cortisol (PC), saliva cortisol (SC), plasma substance P, and plasma haptoglobin concentrations, and the leucocyte mRNA levels of the interleukin-1-alpha (*IL1A*), interleukin-1-beta (*IL1B*), interleukin-1 receptor antagonist (*IL1RN*), and interleukin-6 (*IL6*) genes were analyzed.

**Results:**

Castration decreased (p<0.01) the average daily gain and gain/feed ratio. Castration reduced the time spent eating (p<0.001) and the eating frequency (p<0.01) and increased (p<0.001) the lying frequency. Castration temporarily increased (p<0.05) circulating PC and SC concentrations at 0.5 h after castration. Castration temporarily increased (p<0.05) plasma substance P concentrations at 1 d after castration. Castration increased (p<0.05) plasma haptoglobin concentrations at 1 and 3 d after castration. Castration increased (p< 0.05) leukocyte mRNA levels of the *IL1A*, *IL1B*, *IL1RN*, and *IL6* genes at 6 h after castration.

**Conclusion:**

Castration temporarily induced stress and expression of leucocyte inflammatory cytokine genes in Korean cattle bull calves.

## INTRODUCTION

Castration is a common practice for bulls in several countries, including South Korea. The purpose of castration is to reduce aggressive behavior and improve meat quality [1]. In addition, the meat quality grade of castrated Korean cattle (*Bos taurus coreanae*) is higher than that of bulls [2].

Several studies have reported that castration induces distress, pain, and inflammatory resposnse. For example, knife castration has been reported to raise the plasma cortisol (PC) level [3,4], saliva cortisol (SC) concentration [5], pain indicator—plasma substance P (SP) level [3], and the levels of haptoglobin, which is an indicator of inflammatory reponse [3,6,7].

Research for inflammation related to castration has been conducted for more than 3 days and conducted painful blood sampling several times in that plasma haptoglobin concentration peaked at 2 or 3 d after castration [3,7]. To reduce pain and stress caused by the blood sampling process, an alternative inflammatory biomarker that reaches at the peak earlier than plasma haptoglobin is needed.

Haptoglobin production is induced by the release of several cytokines including interleukin-1 (IL-1) and interleukin-6 (IL-6) from leukocytes at the sites of inflammatory lesions or infections [8]. Leukocyte mRNA levels of cytokines, such as IL-1 and IL-6, may, therefore, be used as earlier indicators of inflammatory responses than haptoglobin concentrations. However, limited information is available regarding the castration effect on leucocyte cytokine gene expression. The objective of this study was to investigate the effect of castration on stress, pain and inflammation indicators, and leucocyte cytokine gene expression for evaluating the level of leucocyte cytokine gene expression as inflammatory indicators after castration.

## MATERIALS AND METHODS

### Animal care

Animal experiments were approved (SNU-180207-4) by the Seoul National University Institutional Animal Care and Use Committee (SNUIACUC) and performed in strict accordance with the recommendations provided by SNUIACUC.

### Calves and housing

Nineteen male Korean cattle calves were used in an experiment conducted at the Seoul National University Animal Farm (Pyeongchang, Korea). Calves were assigned to two groups based on their weight and age: a sham group (control calves; body weight, 254±6.77 kg; age, 8.13±0.11 months; n = 9) and castration group (body weight, 255±9.57 kg; age, 8.17±0.08 months; n = 10). Calves were divided into four pens (four or five heads/pen). Each pen (5×10 m) had sawdust bedding and a self-filling water trough. Calves were individually fed 4.3 kg/d (1.7% body weight) of a commercial young calves concentrate (Cargill, Seongnam, Korea) and 3.6±0.1 kg/d (1.4% body weight) of timothy hay at 08:00, 13:00, and 18:00 h. Concentrate and hay intakes were measured by deducting residual feed from the provided feeds. The composition of the timothy hay was 95.54% dry matter, 11.01% crude protein, and 1.42% crude fat; meanwhile, the composition of the concentrate was 92.0% dry matter, 15.57% crude protein, and 4.08% crude fat (Table 1). The dry matter (method 930.15), crude protein (Kjeldahl N×6.25, method 981.10), ash (method 942.05), ether extract (method 920.39), phosphorus (method 965.17), calcium (method 927.02), and starch (method 948.02) contents of the concentrates and timothy hay were determined using analytical methods provided by the Association of Official Agricultural Chemists [9]. The neutral detergent fiber and acid detergent fiber contents of the timothy hay and the concentrates were analyzed using a sequential method with an ANKOM200 fiber analyzer (ANKOM Technology Corp., Macedon, NY, USA). Details of the method are described in Van Soest et al [10]. Body weight was measured one day before the experiment and at the end of the experiment (14 d).

### Treatments

Calves were castrated in 4 to 5 min, while restrained in a squeeze chute. A small lateral incision on the scrotum was made by a Newberry castration knife (Syrvet Inc., Waukee, IA, USA) to externalize the testicles, and then twisting and cutting of the spermatic cords was achieved using a Henderson castrating tool. Immediately after castration, 7 mL of vitamin K3 (20 mg/mL menadione sodium bisulfite trihydrate; Samyang Anipharm, Seoul, Korea) was delivered into the neck muscle for hemostasis. Sham-castrated animals were treated in the same way as castrated calves and for a similar amount of time, but without making an incision.

### Physiological parameters

Blood and saliva sampling. Blood samples were obtained immediately before castration (0 h) and at 0.5 h, 6 h, 1 d, 3 d, 7 d, and 14 d after castration via jugular venipuncture. Two 10-mL ethylenediaminetetraacetic acid vacutainers (BD, Franklin Lakes, NJ, USA) were used to collect blood for plasma and leukocyte isolation. Plasma was separated from a whole blood sample by centrifugation at 1,500×g at 4°C, and stored at −80°C. Saliva samples were obtained at the same time as the blood sampling, according to the method of Hernandez et al [11]. Briefly, a cotton swab was placed in the mouth of calves for 50 to 60 s. The saliva samples were centrifuged at 4,500×g at 4°C, and stored at −80°C for cortisol analysis. Unfortunately, the amount of saliva collected at 6 h after castration was insufficient for analysis; therefore, saliva data for the period 6 h after castration were not provided.

#### Plamsa analysis

PC and SC levels were analyzed using a SC immunoassay kit according to the specification of kits (Salimetrics LLC, State College, PA, USA). The value for intra- and inter-assay coefficient of variability (CV) of PC was 6.64% and 9.85%, respectively. The intra-assay and inter-assay CV values of SC were 8.84% and 9.13%, respectively. Plasma SP was analyzed using a substance P ELISA kit (Enzo Life Sciences, Farmingdale, NY, USA). The intra-assay CV was 8.42%, whereas the intra-assay CV was 10.6%. Plasma haptoglobin concentrations were analyzed using a bovine haptoglobin enzyme immunoassay kit (GenWay Biotech, San Diego, CA, USA). The intra- and inter-assay CV values were 1.46% and 8.67%, respectively. The analytical methods were verified in our previous study (Park et al [3]).

### Leukocyte isolation

Leukocyte was isolated according to the method of O’Loughlin et al [12]. Briefly, blood leukocytes were isolated using a hypotonic solution and a hypertonic solution. The leukocyte pellets were suspended in 1 mL of TRI Reagent® solution (TRIzol) (Sigma-Aldrich Ireland Ltd., Dublin, Ireland), and stored in a sterile tube at −70°C until RNA isolation.

### Quantitative polymerase chain reaction

Total RNA was isolated from leukocytes using TRIzol reagent according to the supplier’s instructions. The RNA concentration was measured using a nanophotometer. The total RNA integrity was checked by both ethidium bromide staining of the 28S and 18S bands after agarose gel electrophoresis and a bioanalyzer (Agilent Technologies, Santa Clara, CA, USA): an RNA integrity number ≥9.0 was regarded as acceptable. The RNA integrity number of 0 h samples was lower than 8; therefore, the 0 h leukocyte samples were not used in the analysis.

Total RNA was reverse-transcribed into cDNA using the iScript cDNA synthesis kit (Bio-Rad Laboratories Inc., Hercules, CA, USA) according to the manufacturer’s specifications. Reverse transcription was conducted in a 10-μL total reaction volume that contained 2 μg RNA template, 2.5 μL water, 2 μL 5×iScript Reaction Mix, and 0.5 μL iScript reverse transcriptase. The reaction was performed at 25°C for 5 min, at 42°C for 30 min, and at 85°C for 5 min.

The quantitative polymerase chain reaction (qPCR) was performed using QuantiTect SYBR Green RT-PCR Master Mix (Qiagen, Valencia, CA, USA). Primers were designed using integrated DNA technology based on published sequences (Table 2). Primers were designed to include an exon-exon junction for preventing the DNA template amplification. The primer melting temperatures were between 57.0°C and 61.5°C. Therefore, for the amplification of all genes, an annealing temperature of 55°C was used. The qPCR analyses were conducted in a 25-μL total reaction volume that contained 20 ng cDNA, 1.25 μL 10 μM primers, and 12.5 μL SYBR Green RT-PCR Master Mix. The thermal cycler (Rotor-Gene® Q; Qiagen, USA) was programmed to perform thermal cycling: 95°C for 15 min, followed by 40 cycles at 94°C for 15 s, 55°C for 30 s, and 72°C for 30 s. Among three reference genes (18s rRNA, glyceraldehyde 3-phosphate dehydrogenase, and β-actin), the 18s rRNA expression was generally uniform. Therefore, all gene expressions were normalized against the 18s rRNA expression. Because 0 h leukocyte samples were not used, the means of all control samples were used as the calibrator in accordance with the ΔΔCT method [13], enabling the fold changes to be plotted. When the mean of 0.5 h or 14 d control samples were tested as the calibrator, the statistical results were not different from the means of all control samples.

### Statistical analyses

Normality of data was tested using the PROC UNIVARIATE procedure (SAS, version 9.4, SAS Inst. Inc., Cary, NC, USA). Behavior and growth performance results that were not normally distributed were root square +1 transformed. Body weight, average daily gain, feed intake, feed efficiency, and behavioral data were analyzed using the comparing group means model PROC TTEST in SAS. Blood, saliva, and mRNA results, which were not normally distributed, were log-transformed. Blood, saliva, and mRNA results were analyzed using a two-way repeated measures analysis of variance (ANOVA; PROC MIXED procedure in SAS). The fixed effects were time, castration, and their interactions. Differences between castration and non-castration groups at each time point were compared using the model PROC TTEST in SAS. The correlations between the concentration of blood and saliva parameters and relative cytokine mRNA levels were determined using the PROC CORR procedure in SAS. Significance was determined by p≤0.05 and tendency was revealed by 0.05<p≤0.10.

## RESULTS AND DISCUSSION

### Plasma cortisol and saliva cortisol concentrations

**Figure 1:**
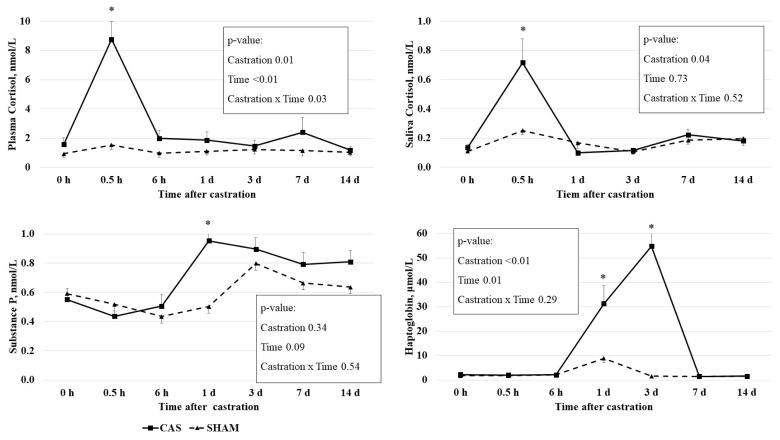
Effect of castration on plasma concentrations of cortisol, substance P, and haptoglobin and saliva cortisol concentrations in Korean cattle calves. Male calves were allocated into two groups: CAS = castration (n = 10); SHAM = sham (n = 9). Values represent the mean+standard error of the mean. The p-values indicate results of two-way repeated measures analysis of variance analysis. An asterisk (*) indicates that the values differ between castration and sham group at each time point (p<0.05, t-test).

The castration effects on the PC and the SC concentrations are presented in Figure 1. PC concentration had a castration × time interaction effect (p = 0.03), time effect (p<0.01), and castration effect (p = 0.01). Castration increased the PC concentrations at 0.5 h, but not at other times. SC concentration had a castration effect (p = 0.04): castration increased SC concentrations at 0.5 h, but not at other times. The elevated PC and SC concentrations returned to levels similar to those of 0 h at 6 h or 1 d after castration.

Blood cortisol was considered as an indicator of castration stress in cattle [1]. Previous studies have also reported increases in PC concentrations in castrated calves within a short time period, e.g., a PC increase at 0.5 h after surgical castration in 6.3-month-old calves [3] and a PC increase at 0.5 h after surgical castration in 3-month-old calves [4]. The cortisol-based stress response induced by castration can be easily eliminated through the rapid clearance of cortisol from the bloodstream [14].

Consistent with our results, castration increased the SC concentrations at 1 and 2 h after surgical castration in 4-month-old calves [15] and at 1 h after band castration in 219-d-old calves [5].

The dynamic changes of both PC and SC concentrations after castration were similar, and a positive correlation between PC and SC concentrations (r = 0.69; p<0.01) was observed (Table 3; Figure 1). To our knowledge, there has been no study investigating the relationship between PC and SC after the castration of bull calves. Previous studies have found that the SC concentration reflected the blood cortisol concentration when cattle were injected with adrenocorticotropic hormone [10,16]. Vining and McGinley [17] reported that blood free cortisol is diffused passively to saliva from the acinar cells surrounding the lumen of the salivary gland. Taken together, our study and others have demonstrated that SC may reflect PC under castration stress. Our findings suggest that an SC assessment could be a better method for measuring cortisol than a PC assessment because the collection of saliva samples is much easier and causes less stress than the collection of blood samples from animals.

### Plasma substance P

Plasma SP concentrations have been used to evaluate muscle pain, soft tissue injuries in humans, and the castration pain of calves [3,18]. A tendency (p = 0.09) of time effect on the plasma SP concentration was observed. The SP concentration was higher (p<0.05) in castrated calves than in non-castrated calves at 1 d after castration, but not at other time points (Figure 1).

Several studies have revealed variations in castration effects on the SP concentration in cattle. The SP concentrations were found to be higher in 4- to 6-month-old castrated calves than non-castrated calves 4 h after castration [18]. Park et al [3] observed a temporal induction of SP at 6 h after castration in 6-month-old castrated calves compared to non-castrated calves. However, the SP concentrations in castrated calves were not significantly different from those in sham calves at 1 and 2 h or 7 d after castration in 1-week-, 2-month-, and 4-month-old calves [15]. In this study, SP concentrations were higher at 1 d after castration in 8-month-old castrated calves. The SP concentrations in castrated calves were declined by 3 d after castration [19], leading to the same authors warning of the potential weaknesses of using SP as an indicator of pain from surgical castration. The differential responses of the SP concentration upon castration among several studies may be due to the differences in animal age and time of observation. Collectively, these results reveal the limitation of using the SP concentration as an indicator of the pain resulting from castration.

### Plasma haptoglobin

Haptoglobin concentrations had a time (p = 0.01) effect and a castration effect (p<0.01). Castration increased haptoglobin concentrations at 1 d (p<0.05) and 3 d (p<0.01), but not at other time points (Figure 1).

Haptoglobin, which is produced by hepatocytes, is participated in inflammatory process, inducing anti-inflammatory processes [20]. Haptoglobin is involved in defense systems against acute changes such as infection, damage, injury, or stress [21]. It has been suggested that haptoglobin can be used as an inflammatory indicator in cattle [22]. In this study, we observed an increase in the haptoglobin concentration 1 and 3 d after castration. Consistent with our results, castration increased haptoglobin concentrations at 1 d and 3 d in 6.3-mo- [3] and 7-mo-old [23].

### Leukocyte cytokine gene expression

**Figure 2:**
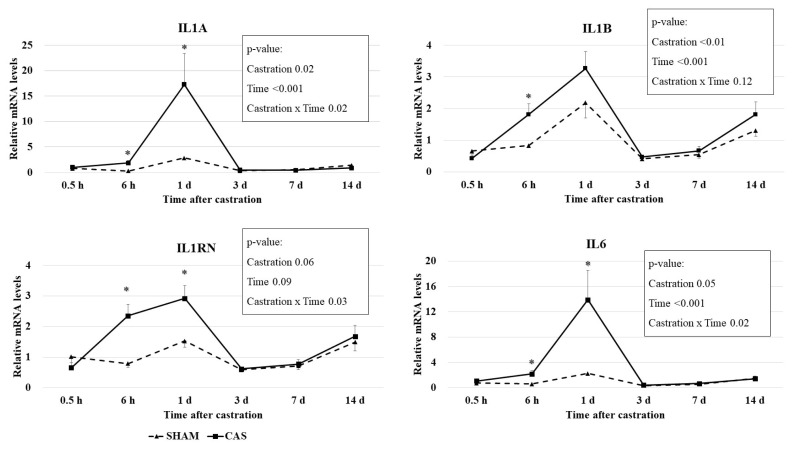
Effect of castration on leukocyte cytokine gene expression in Korean cattle calves. Male calves were allocated into two groups: CAS = castration (n = 10); SHAM = sham (n = 9). Relative gene expression levels were normalized to average mRNA levels of sham groups of all times. IL6, interleukin 6; IL1A, interleukin 1 alpha; IL1B, interleukin 1 beta; IL1RN, interleukin 1 receptor antagonist. The p-values indicate results of two-way repeated measures analysis of variance analysis. An asterisk (*) indicates that the values differ between castration and sham group at each time point (p<0.05, t-test).

Castration effects on relative mRNA levels of cytokine genes in blood leukocytes are presented in Figure 2. Castration × time interaction effects (p<0.05) were observed for IL6, interleukin-1 alpha (IL1A), and interleukin-1 receptor antagonist (IL1RN) mRNA levels. The mRNA levels of the *IL6*, *IL1A*, and *IL1B* genes had a time effect (p<0.001) and a castration effect (p≤0.05) based on the two-way repeated measures ANOVA. Castration increased (p<0.05) the expression levels of the *IL6*, *IL1A*, and *IL1RN* genes at 6 h and 1 d, but not at other times (Figure 2). Castration increased (p<0.05) the expression levels of the *IL1B* gene at only 6 h.

Cytokines are predominantly made by leukocytes, including lymphocytes and granulocytes. IL-1α, IL-1β, and IL-6 promote inflammation and are called proinflammatory cytokines. IL-6 secretion is induced by a variety of stimulating signals, including lipopolysaccharide, IL-1, and TNF-α, and platelet-derived growth factor [24]. IL-1ra suppresses the activity of IL-1 and is an anti-inflammatory cytokine. A previous study reported that the relative mRNA quantity of the interferon gamma, *IL6*, interleukin-10, and *TNF-α* genes in leukocytes was not different between banding- or burdizzo-castrated calves and non-castrated calves [25]. Differences in castration methods may affect this discrepancy. Pang et al [25] used the burdizzo and banding castration methods, which do not make an incision in the scrotum, and thus might not cause inflammation. In our study, we used knife castration, which made wounds on the stratum and ductus deferens. Inflammatory responses may be induced due to these wounds, resulting in the induction of cytokine gene expression.

Leukocyte mRNA levels of IL1A (r = 0.96; p<0.01) and IL1B (r = 0.55; p<0.01) were positively correlated with leukocyte mRNA levels of IL6 (Table 3). A correlation between IL-1β and IL-6 was observed in a previous study [26]. These positive correlations may reflect IL-1 induction through activation of IL-6.

Leukocyte mRNA levels of IL1RN were positively correlated with the leukocyte mRNA levels of IL6 (r = 0.67; p<0.01) and IL1B (r = 0.83; p<0.01; Table 3). IL-1ra, a product of the *IL1RN* gene, suppresses the action of IL-1α and IL-1β by inhibiting the binding of IL-1α and IL-1β to interleukin-1 receptor [27]. The IL-1ra levels are raised in pateints suffering from infection or inflammation (reviewed by [28]). Several studies have reported a simultaneous elevation in both IL-1ra and IL-6 or IL-1β. For example, increased IL-1ra levels following IL-6 infusion have been reported in healthy males [29]. Given the importance of a balance between IL-1 and IL-1ra in inhibiting the occurrence of inflammatory diseases [30], IL-1ra expression may have increased to regulate the increased IL-1 levels caused by castration. Haptoglobin level and the cytokines mRNA levels were not correlated each other. Correlation analysis was conducted using the values of two variables at each time point. However, given that IL-1 and IL-6 stimulate haptoglobin production, mRNA levels of IL1 and IL6 increased before plasma haptoglobin levels increased.

This was the first study to show a relationship between knife castration and leukocyte cytokine mRNA levels. Increased IL1A, IL1B, IL6, and IL1RN mRNA levels were observed 1 d after castration, while haptoglobin levels peaked after 3 d. Our results suggest that the haptoglobin response can be driven by the early expression of *IL1A*, *IL1B*, *IL6*, and *IL1RN* genes as innate inflammatory indicators in castrated calves.

## CONCLUSION

Surgical castration increased stress, pain, inflammation indicators, and the mRNA levels of *IL1A*, *IL1B*, *IL6*, and *IL1RN* genes in the leukocytes in the early post-castration period. Our study suggests that the mRNA levels of some innate types of cytokine can be used to determine inflammatory and stress responses for calves after castration. This study also suggests that alleviation strategies for stress, pain, and inflammation are needed simultaneously after surgical castration.

## Figures and Tables

**Table 1:** Ingredients and chemical composition of experimental feeds

Ingredient	Percentage	Chemical composition	Percentage
	
Concentrate ingredients (DM basis)		DM	92.0
Ground corn	15.82	Crude ash	7.145
Ground wheat	18.00	CP	15.57
Salt	0.88	EE	4.08
Molasses	5.50	ADF	11.49
Wheat bran	3.00	NDF	27.495
Corn flour	5.00	NFC^2)^	45.71
Rice bran	3.00	Calcium	1.21
Ammonium chloride	0.15	Phosphate	0.47
Condensed molasses solubles	1.50	TDN^3)^ (%)	88.705
Cottonseed hulls	1.50	DE^4)^ (Mcal/kg)	3.91
Porphyry	2.00	ME^5)^ (Mcal/kg)	3.46
Rapeseed meal	2.22	Timothy composition	
Limestone	3.30	DM	95.54
Corn gluten feed	8.50	Crude ash	6.77
Dried distilled grain solubles	9.38	CP	11.01
Palm kernel meal	10.00	EE	1.42
Copra meal	10.00	Calcium	0.28
Mineral/vitamin premix^1)^	0.25	Phosphate	0.275
Total	100.00	ADF	35.68
		NDF	63.05

DM, dry matter; CP, crude protein; EE, ether extract; ADF, acid detergent fiber; NDF, neutral detergent fiber; NFC, non fiber carbohydrate; TDN, total digestible nutrient; DE, digestible energy; ME, metabolizable energy.

1) Mineral and vitamin premix contained 13.2 g Fe, 4.4 g Mn, 2.2 g Cu, 0.44 g I, 4.4 g Zn, 0.44 g Co, 530,000 IU vitamin D_3_, 2,650,000 IU vitamin A, 10 g Niacin, 1,050 IU vitamin E per kg of additive (Grobic-DC, Bayer Health Care, Leverkusen, Germany).

2) NFC (%) = 100–(CP+EE+ash+NDF).

3) TDN (%) = NFC+CP+((EE–1)×2.25)+NDF–7 (NRC, 2016).

4) DE (Mcal/kg) = 0.04409×TDN (NRC, 2016).

5) ME (Mcal/kg) = (1.01×(DE)–0.45)+0.0046×(EE–3) (NRC, 2016).

**Table 2:** Primer sequences and amplicon information for real-time polymerase chain reaction

Gene name (abbreviation)	Sequences (5′-3′)	Product size (bp)
Interleukin 1 alpha (*IL1A*)	F: ATGACCTGGAAGCCATTGCC	120
	R: TGCATTCCTGGTGGATGACTC	
Interleukin 1 beta (*IL1B*)	F: GTCATCGTGGCCATGGAGAA	143
	R: GCGTCACACAGAAACTCGTC	
Interleukin 6 (*IL6*)	F: CTCTCATTAAGCGCATGGTCG	142
	R: AAGCATCCGTCCTTTTCCTCC	
Interleukin 1 receptor antagonist (*IL1RN*)	F: TGGCCTGCGTAAAATCTGGA	111
	R: CGAAGCGGATGAAGGCAAAG	
18S ribosomal RNA (*18S rRNA*)	F: GGCCGTTCTTAGTTGGTGGA	150
	R: TCAATCTCGGGTGGCTGAAC	

**Table 3:** Pearson correlation coefficients among plasma and saliva parameters and blood leukocyte cytokine mRNA levels in Korean cattle calves^1)^

Item (sample number)^2)^	Plasma and saliva items				mRNA levels			
	
	PC (133)	SC (114)	SP (133)	HP (133)	IL1A (114)	IL1B (114)	IL1RN (114)	IL6 (114)
PC (133)	-							
SC (114)	0.69^**^	-						
SP (133)	−0.06	−0.18	-					
HP (133)	−0.06	−0.11	0.25^**^	-				
IL1A (114)	−0.03	−0.10	0.17	−0.10		-		
IL1B (114)	−0.12	−0.15	0.04	−0.07		0.55^**^	-	
IL1RN (114)	−0.06	−0.08	0.14	−0.10		0.48^**^	0.83^**^	-
IL6 (114)	−0.02	−0.10	0.14	−0.13		0.96^**^	0.55^**^	0.67^**^

PC, plasma cortisol; SC, saliva cortisol; SP, plasma substance P; HP, plasma haptoglobin; IL1A, interleukin-1 alpha; IL1B, interleukin-1 beta; IL1RN, interleukin-1 receptor antagonist; IL6, interleukin-6.

1) Statistical significance:

** p<0.01.

2) Numerical values in parenthesis indicate the number of samples used as follows. A total 133 samples of each of PC, SP, and HP were used in the correlation analysis [19 animals×7 time points (0 h, 0.5 h, 6 h, 1 d, 3 d, 7 d, and 14 d)]. A total of 114 samples of SC were used in the correlation analysis [19 animals × 6 time points (0 h, 0.5 h, 1 d, 3 d, 7 d, and 14 d)]. A total of 114 samples of each of mRNA level for the four genes were used in the correlation analysis [19 animals × 6 time points (0.5 h, 6 h, 1 d, 3 d, 7 d, and 14 d)]. The number of actual correlation pairs were 133, 114, or 95, depending on the two parameters being compared.
